# The complete mitochondrial genome of *Cynops cyanurus chuxiongensis* (Caudata: Salamandridae)

**DOI:** 10.1080/23802359.2019.1698987

**Published:** 2019-12-13

**Authors:** Lin Cui, Fuyao Han, Diyan Li, Xiaolan Fan, Mingyao Yang, Deying Yang, Qingyong Ni, Yan Li, Yongfang Yao, Huailiang Xu, Bo Zeng, Ying Li, Feida Sun, Dongru Zhang, Mingwang Zhang

**Affiliations:** a College of Animal Science and Technology, Sichuan Agricultural University, Chengdu, PR China;; b Animal Genetic Resources Exploration and Innovation Key Laboratory of Sichuan Province, Sichuan Agricultural University, Chengdu, PR China;; c College of Life Science, Sichuan Agricultural University, Ya’an, PR China;; d State Key Laboratory of Genetic Resources and Evolution, Kunming Institute of Zoology, Chinese Academy of Sciences, Kunming, China

**Keywords:** *Cynops cyanurus chuxiongensis*, mitogenome, Salamandridae, phylogenetic relationship

## Abstract

*Cynops cyanurus chuxiongensis* is distributed in Yunnan plateau. In this paper, we sequenced and determined the complete mitochondrial genome of *C. c. chuxiongensis.* The assembled mitogenome is 16,465 bp in length and encoding 13 protein-coding genes (PCGs), 22 transfer RNA (tRNA) genes, two ribosomal RNA genes (12S rRNA and 16S rRNA), and one control region (D-loop). The phylogenetic trees indicated *C. c. chuxiongensis* (KY418068) has the closest relationship with *C. orientalis* + *C. orphicus*, and clustered within the clade of genus *Cynops.*

Chuxiong Fire-bellied Newt (*Cynops cyanurus chuxiongensis)* is endemic to China and only distributed in the mountainous areas of Chuxiong, Yunnan province at an altitude of 2100–2400 m (Fei and Ye 1983; Fei et al. 2012). Unfortunately, it has been listed as near threatened (NT) species in 2004 (Wang and Xie 2004; Jiang et al. 2016). Compared with individual mitochondrial regions, the mitogenome has been used as a well-accepted molecular tool to improve phylogenetic inference by providing stronger branch support. In this study, we sequenced and characterized the mitogenome of *C. c. chuxiongensis* and investigated the phylogenetic relationships among the newts.

The sample was collected in Wuding county (25°32′28.57″N, 102°22′44.74″E), Yunnan Province, China, then preserved in 99.9% ethanol and deposited at the Museum of Sichuan Agricultural University (Accession number: SAU2014005). Genomic DNA was extracted from the tail tip tissue using the Ezup pillar genomic DNA extraction kit (Sangon Biotech, Shanghai, China). 13 pairs of oligonucleotide primers published by (Zhang et al. 2008) were used to amplify and sequence the contiguous and overlapping fragments that covered the entire mitogenome. Finally, we aligned and assembled the entire mitogenome of *C*. *c*. *chuxiongensis* using the DNASTAR 6.0. The new mitogenome sequence determined here with annotations has been submitted to GenBank (KY418068).

The mitogenome of *C. c. chuxiongensis* is 16,465 bp in length and encodes 37 genes, including 13 protein-coding genes (PCGs), two ribosomal RNA (12S rRNA and 16S rRNA), 22 transfer RNA genes (tRNAs), one mitochondrial control region (CR or D-loop), and one non-coding region (NC). The base composition was 32.4% A, 27.7% T, 24.3% C, and 15.6% G, demonstrating a bias of higher AT content (60.1%) than GC content (39.9%). The positions of all genes were predicted using the MITOS Web Server (Matthias et al. 2013). Except for eight tRNA genes (tRNA^Gln^, tRNA^Ala^, tRNA^Asn^, tRNA^Cys^, tRNA^Pro^, tRAN^Ser(UCN)^, tRNA^Tyr^, and tRNA^Glu^), all the rest of tRNA genes are encoded on the H-strand. Most of the PCGs begin with an initiation ATG codon, only COI starts with GTG. Besides, eight PCGs end with complete TAA, TAG, or AGA stop codon, whereas the rest four genes (COII, COIII, ND4, and Cyt*b*) are terminated by incomplete TA(A) or T(AA). The length of most genes is consistent with their counterpart in other Salamandridae species (Chen et al. 2016). The non-coding region (NC) between the tRNA^Pro^ and tRNA^Phe^ genes in *C. c chuxiongensis* is 368 bp long while in other salamander species is 160–163 bp long (Zhang et al. 2008; Chen et al. 2016).

We inferred phylogenetic relationships of *C. c. chuxiongensis* with the concatenated dataset of all 13 PCGs by the maximum-likelihood (ML) using IQTREE-1.6.8 with ultrafast bootstrap (UFBoot) (Thi et al. 2017). The final concatenated alignment consists of 11,460 bp for 21 species from seven genera (*Tylototriton*, *Triturus*, *Cynops*, *Laotriton*, *Pachytriton*, *Paramesotriton*, and *Alligatorinae*), and we select *Alligator mississippiensis* as an outgroup. The phylogenetic trees based on these mitogenomes indicated *C. c. chuxiongensis* has the closest relationship with *C. orientalis* + *C. orphicus*, and clustered within clade of genus *Cynops* (Figure 1). This newly sequenced complete mitogenome would contribute to further investigations of molecular evolution and conservation of genus *Cynops*.

**Figure 1.**
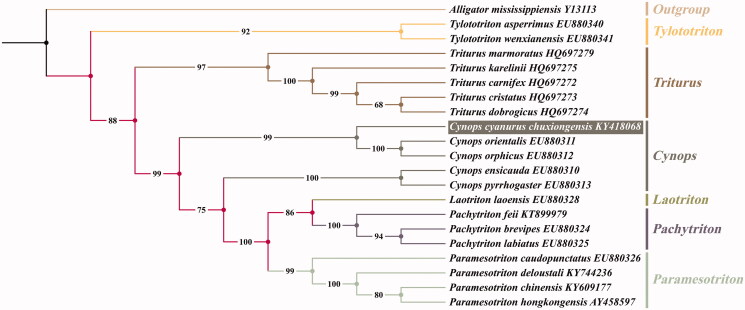
Maximum-likelihood (ML) phylogenetic tree yielded by IQTREE for family Salamandridae based on 13 protein-coding genes from 21 species. Bootstrap values are shown in the middle of the branches connected to the nodes. *Alligator mississippiensis* was chosen as an outgroup.

## References

[CIT0001] Chen X, He K, Ding ZL, Li GM, Adeola AC, Murphy R, Wang WZ, Zhang YP. 2016. An improved de novo pipeline for enrichment of high diversity mitochondrial genomes from Amphibia to high-throughput sequencing. BioRxiv, 080689, available at doi:10.1101/080689

[CIT0002] Fei L, Ye C. 1983. A new subspecies of *Cynops cyanurus* from Chuxiong. Yunnan (Caudata: Salamandride).Acta Herpetol Sin. 2(4):55–58.

[CIT0003] Fei L, Ye C, Jiang J. 2012. Colored Atlas of Chinese amphibians and their distributions. Chengdu: Sichuan Publishing House of Science and Technology.

[CIT0004] Jiang J, Xie F, Zang C, Cai L, Li C, Wang B, Li J, Wang J, Hu J, Wang Y, et al. 2016. Assessing the threat status of amphibians in China. Biodivers Sci. 24(5):588–597.

[CIT0005] Matthias B, Alexander D, Frank J, Fabian E, Catherine F, Guido F, Joern P, Martin M, Stadler PF. 2013. MITOS: improved de novo metazoan mitochondrial genome annotation. Mol Phylogent Evol. 69(2):313–319. 10.1016/j.ympev.2012.08.023 22982435

[CIT0006] Thi HD, Olga C, Arndt vH, Quang MB, Sy VL. 2017. UFBoot2: improving the ultrafast bootstrap approximation. Mol Biol Evol. 2:2. 10.1093/molbev/msx281 PMC5850222 29077904

[CIT0007] Wang S, Xie Y. 2004. Chinese Species Red List (Vol. 1 Red List). Beijing (People's Republic of China): Higher Education Press.

[CIT0008] Zhang P, Papenfuss TJ, Wake MH, Qu L, Wake DB. 2008. Phylogeny and biogeography of the family Salamandridae (Amphibia: Caudata) inferred from complete mitochondrial genomes. Mol Phylogent Evol. 49(2):586–597. 10.1016/j.ympev.2008.08.020 18801447

